# Screening tool for diagnosis childhood obesity: percent weight for height vs body mass index

**DOI:** 10.1186/1687-9856-2013-S1-P101

**Published:** 2013-10-03

**Authors:** Voraluck Phatarakijnirund, Taninee Sahakitrungruang, Vichit Supornsilchai, Suttipong Wacharasindhu

**Affiliations:** 1 Pediatric Endocrine Unit, Department of Pediatrics, Faculty of Medicine, Chulalongkorn University, Bangkok, 10330 Thailand; 2 Growth and Growth Monitoring Center, King Chulalongkorn Memorial Hospital, Bangkok, 10330 Thailand

## Background

Childhood obesity is a worldwide epidemic problem and it prevalence has been increasing over the time, however, there is no best single standard criteria for screening obesity in population. In this study, we aim to assess the prevalence of obesity in primary school children by using percent weight for height (PWH) criteria compare with body mass index (BMI) curve from International Obesity Task Force (IOTF) and study the correlation between these two criteria.

## Method

A cross-sectional study was performed during July 2009 - January 2010 in grade 3-6 children from 3 primary schools which was selected by stratified proportionate sampling. The program consists of measuring of individual height and weight in all children and these data were used to calculate percent weight for height (PWH) and body mass index (BMI). The correlation between these two criteria was assessed by using Pearson Correlation coefficient.

## Result

Total number of subjects was 1,223 (637 boys and 586 girls), age 9.68 + 1.2 years. The prevalence of childhood obesity assessed by PWH (overall rate: 15.3%, boy: 19.3%, girl: 10.9%) was higher than using BMI (overall rate: 11.9%, boy: 15.7%, girl: 7.8%). When the rate of childhood obesity was compared by age group, the prevalence of obesity in prepubertal children by PWH criteria was higher when compare to BMI criteria. By contrast, in pubertal children the prevalence of obesity was higher when using BMI criteria. The correlation between PWH and BMI criteria was fair (r = 0.66) but increasing according to age(r = 0.61 at age of 8 to 0.74 at age of 12 year)

## Conclusion

The prevalence of childhood obesity using PWH and BMI criteria from IOTF was significantly different. The correlation between these two parameters was fair. Further studies would be needed to determine the clinical validity of these parameters as a tool to screen and provide intervention for childhood overweight.

